# Fmrp regulates neuronal balance in embryonic motor circuit formation

**DOI:** 10.3389/fnins.2022.962901

**Published:** 2022-11-03

**Authors:** Chase M. Barker, Kaleb D. Miles, Caleb A. Doll

**Affiliations:** ^1^Section of Developmental Biology, Department of Pediatrics, Children’s Hospital Colorado, University of Colorado School of Medicine, Aurora, CO, United States; ^2^Biomedical Sciences and Biotechnology Program, Graduate School, University of Colorado, Aurora, CO, United States

**Keywords:** Fragile X syndrome, cell fate specification, synapse development, motor circuits, motor neuron development, GABAergic interneurons

## Abstract

Motor behavior requires the balanced production and integration of a variety of neural cell types. Motor neurons are positioned in discrete locations in the spinal cord, targeting specific muscles to drive locomotive contractions. Specialized spinal interneurons modulate and synchronize motor neuron activity to achieve coordinated motor output. Changes in the ratios and connectivity of spinal interneurons could drastically alter motor output by tipping the balance of inhibition and excitation onto target motor neurons. Importantly, individuals with Fragile X syndrome (FXS) and associated autism spectrum disorders often have significant motor challenges, including repetitive behaviors and epilepsy. FXS stems from the transcriptional silencing of the gene Fragile X Messenger Ribonucleoprotein 1 (FMR1), which encodes an RNA binding protein that is implicated in a multitude of crucial neurodevelopmental processes, including cell specification. Our work shows that Fmrp regulates the formation of specific interneurons and motor neurons that comprise early embryonic motor circuits. We find that zebrafish *fmr1* mutants generate surplus ventral lateral descending (VeLD) interneurons, an early-born cell derived from the motor neuron progenitor domain (pMN). As VeLD interneurons are hypothesized to act as central pattern generators driving the earliest spontaneous movements, this imbalance could influence the formation and long-term function of motor circuits driving locomotion. *fmr1* embryos also show reduced expression of proteins associated with inhibitory synapses, including the presynaptic transporter vGAT and the postsynaptic scaffold Gephyrin. Taken together, we show changes in embryonic motor circuit formation in *fmr1* mutants that could underlie persistent hyperexcitability.

## Introduction

The integration of distinct cell types into neural circuits occurs during precise developmental windows. In zebrafish locomotive circuits, early-born primary motor neurons (MNs) and interneurons (INs) connect to form rudimentary networks that drive early spontaneous behavior (Saint-Amant and Drapeau, 2001). INs are crucial for this process, as isolated MN clusters must develop synchrony with adjacent MN groups to achieve coordinated locomotion by driving muscle contractions throughout the spine (Cazalets et al., 1992; Tresch and Kiehn, 2000). In more mature circuits, spinal INs provide excitatory and inhibitory modulation of motor neuron output to drive refined locomotion (McLean et al., 2007, 2008; Kimura et al., 2013; Callahan et al., 2019). Less is known about IN function in embryogenesis, though some INs appear to function as central pattern generators, synchronizing MN output in adjacent hemisegments to drive early spontaneous behavior (Saint-Amant and Drapeau, 2000, 2001; Warp et al., 2012). Importantly, changes in the relative amount of synchronizing IN output could profoundly influence nascent neural circuit formation and long-term motor function.

Fragile X syndrome (FXS) is the most common heritable cause of intellectual disability, and individuals with FXS commonly have symptoms consistent with hyperexcitable motor behavior, including repetitive movements and epilepsy (Berry-Kravis, 2002; Oakes et al., 2016). Many vertebrate models of FXS show hyperexcitable motor behavior, including zebrafish *fmr1* mutants at both larval and adult stages (Kim et al., 2014; Shamay-Ramot et al., 2015). Although FXS appears to be rooted in neurodevelopmental mechanisms, the bulk of FXS research has focused on synaptic dysfunction in established neural circuits. It has been difficult to pinpoint the pathogenesis of FXS due to the widespread influence of Fragile X Messenger Ribonucleoprotein (FMRP; Fmrp in zebrafish) on synapse formation and function (Huber et al., 2002; Todd et al., 2003; Dictenberg et al., 2008; Doll et al., 2017). A leading hypothesis is that FXS symptoms are rooted in altered excitatory and inhibitory (E/I) balance, including diminished inhibitory gamma-aminobutyric acid (GABA) signaling in mature FXS circuits (Fatemi et al., 2009; Hashemi et al., 2017; Goel et al., 2018). However, there is also evidence that GABAergic signaling in embryogenesis is altered in the absence of FMRP, such that GABA remains depolarizing at later developmental stages (He et al., 2014; Zhang et al., 2022). It is not yet known when motor defects first develop in FXS; we hypothesize that Fmrp is required in early embryogenesis to proportionally generate specialized interneuron and motor neuron subtypes that provide balanced excitation and inhibition in developing locomotive circuits.

The specification of distinct cell types from common progenitor domains requires the regulation of unique gene expression profiles that are tailored to a specific lineage. FMRP regulates a diverse catalog of genes and is classically associated with synaptogenesis and neural plasticity (Dictenberg et al., 2008; Darnell et al., 2011; Deng et al., 2013; Doll and Broadie, 2015; Doll et al., 2017; Maurin et al., 2018). Fmrp also regulates cell fate decisions in embryogenesis: for example, Fmrp regulates the balance of cells generated from the motor neuron progenitor domain (pMN) in the ventral spinal cord, including MNs and oligodendrocyte lineage cells (Doll et al., 2021). pMN progenitors also produce specialized spinal interneurons that modulate MN output to drive muscle contractions (Park et al., 2004; Warp et al., 2012; Svara et al., 2018). We find that zebrafish *fmr1* mutants generate excess GABAergic INs in the ventral spinal cord and show reduced inhibitory synaptogenesis in embryonic stages, at a developmental stage when motor behavior initiates. Fate mapping reveals excess INs are early-born ventral lateral descending (VeLD) cells born in the pMN domain alongside primary motor neurons. Given proposed roles for VeLD neurons as central pattern generators driving the earliest spontaneous contractions (Warp et al., 2012), our work presents a new hypothesis to help explain the origins of hyperexcitability in FXS.

## Materials and methods

### Zebrafish lines and husbandry

The Institutional Animal Care and Use Committee at the University of Colorado School of Medicine approved all animal work, which follows the US National Research Council’s Guide for the Care and Use of Laboratory Animals, the US Public Health Service’s Policy on Humane Care and Use of Laboratory Animals, and Guide for the Care and Use of Laboratory Animals. Larvae were raised at 28.5°C in embryo medium and staged as hours (hpf) according to morphological criteria (Kimmel et al., 1995). Zebrafish lines used in this study included *fmr1^*hu*2787^* (den Broeder et al., 2009), *Tg(olig2:EGFP)^*vu*12^* (Shin et al., 2003), *Tg(slc17a6b:EGFP)^*zf*139^* (Miyasaka et al., 2009), and *Tg(mnx1:EGFP)^*ml*2^* (Flanagan-Steet et al., 2005). All mutants were maternal-zygotic *fmr1^*hu*2787^*. Genotyping for *fmr1^*hu*2787^* was performed as previously described (Ng et al., 2013). As zebrafish sex determination does not occur until juvenile stages (Uchida et al., 2002), we were unable to determine the sex of the embryos in our experiments.

### Imaging and analysis

We acquired images on a Zeiss LSM 880 or a Zeiss Cell Observer SD 25 spinning disk confocal system (Carl Zeiss). Images were captured with Zen software (Carl Zeiss), then processed and analyzed using Fiji/ImageJ. Live images in Figure 5 were captured as previously described (Doll et al., 2020).

### Fluorescent *in situ* RNA hybridization

The probes for zebrafish *gata3* and *lhx3* were designed and synthesized by the manufacturer for use with the B2 and B3 amplifiers and B2–546 nm and B3–647 fluorophores, respectively (Molecular Instruments). Fluorescent *in situ* hybridization (FISH) procedure was guided by the *in situ* hybridization chain reaction protocol for whole-mount zebrafish (Molecular Instruments v3.0; Choi et al., 2018). Embryos/larvae at indicated timepoints were fixed in 1 mL of 4% paraformaldehyde (PFA)/1xPBS for 24 h at 4°C. Samples underwent 3 × 5-min washes with 1 mL of 1 × phosphate-buffered saline (PBS) to stop the fixation, followed by 1 mL 4 × 10-min and 1 × 50-min 100% MeOH washes. Samples were stored overnight at −20°C, then transferred to a 1.5 mL Eppendorf tube and then proceeded through a series of 1 mL MeOH/0.1% Tween 20/1xPBS (PBSTw) washes for 5 min each at room temperature as follows. 1 × 75% MeOH/25% PBSTw, 1 × 50% MeOH/50% PBSTw, 1 × 25% MeOH/75% PBSTw, and 5 × 100% PBSTw. Samples were treated with Proteinase K (24 hpf at 1:1000 for 5 min; 48 hpf at 1:200 for 8 min), immediately washed twice with PBSTw (1 mL each) without incubation, postfixed with 1 mL of 4% PFA for 20 min at room temperature on a rocker, then washed for 5 × 5-min with 1 mL of PBSTw. For the detection stage, 500 μL of pre-warmed probe hybridization buffer was added for 30 min at 37°C. Then the pre-hybridization solution was removed and 500 μL of probe solution (2 μL of probe/500 μL of pre-warmed probe hybridization buffer) was added and incubated overnight (12–16 h) at 37°C. The next day, probe wash buffer was warmed to 37°C before starting washes. Samples underwent 4 × 15-min washes with 500 μL of probe wash buffer at 37°C followed by 2 × 5-min washes with 5 × 1X saline sodium citrate (SSC), with 0.1% Tween (SSCT) at room temperature. Before the next wash, the amplification buffer was equilibrated to room temperature, then samples were pre-amplified with 500 μL of amplification buffer for 30 min at room temperature. During the pre-amplification step, 30 ρmol of hairpin h1 and 30 ρmol of hairpin h2 were separately prepared by snap cooling 10 μL of 3 μM stock at 95°C for 90 s, and then allowed to cool to room temperature in a dark drawer for 30 min. The hairpin solution was then prepared by adding snap-cooled h1 hairpins and snap-cooled h2 hairpins to 500 μL of amplification buffer at room temperature. After the completion of the pre-amplification step, the pre-amplification solution was removed, 125 μL of hairpin solution was added, per 1.5 mL tube containing ∼12 embryos/larvae, which were incubated overnight (12–16 h) in the dark at room temperature. The following day the excess hairpins were removed by a series of washes with 500 μL of 5 × SSCT for 2 × 5-min, 2 × 30-min, and 1 × 5-min at room temperature. Samples were post-fixed in 1 mL of 4% paraformaldehyde/1xPBS for 20 min on a rocker. Then two washes were performed with 1 mL of 1xPBS diethyl pyrocarbonate (DEPC) with no incubation time.

### Immunohistochemistry

Embryos were fixed in 4% paraformaldehyde/1xPBS and rocked overnight at 4°C. Samples were rinsed in 1xPBS, then embedded in 1.5% agar/30% sucrose blocks and immersed in 30% sucrose overnight. Blocks were frozen on dry ice, then 20 μm transverse sections from the spinal trunk at the level of the yolk tube were taken with a cryostat microtome and collected on polarized slides. Slides were mounted in Sequenza racks (Thermo Scientific, Waltham, MA, USA), washed twice for 5 min in 0.1% Triton/1xPBS (PBSTx) to rehydrate samples, blocked 1 h in 2% goat serum/2% bovine serum albumin/PBSTx, 200 μL per slide. Samples were then incubated in primary antibodies: rabbit α-GABA (1:500; Sigma, A2052; Pedroni and Ampatzis, 2019); mouse α-Gephyrin (1:500; Synaptic Systems; Marisca et al., 2020); and rabbit α-vGAT (1:500; Synaptic Systems; Pedroni and Ampatzis, 2019). Sections were washed 6 × 15-min in PBSTx, then incubated 2 h at room temperature in 200 μL of secondary antibody (1:500; in block): Alexa Fluor goat α-rabbit 488 (A-11008; Invitrogen), Alexa Fluor goat α-mouse 568 (A-11004; Invitrogen). Sections were washed for 1 h (4 × 15-min washes) in PBSTx, incubated with 200 μL of 4′,6-diamidino-2-phenylindole (DAPI) (1:1000 in PBSTx) for 5 min, washed for 30 min (2 × 15 min washes) in PBSTx, then mounted in Vectashield (Vector Laboratories, H-1000-10). For FISH/IHC combination experiments, wholemount embryos immediately underwent the immunohistochemistry (IHC) protocol after hairpin washes and post-fixation.

### Quantification and statistical analyses

For all cell counts, the investigator was blind to genotype. For IHC, DAPI, and GABA/Islet channels were used to confirm cell number in each section. For FISH, DAPI, and *gata3* or *lhx3* were used to confirm cell number in acquired *z*-stacks. For quantification of sectioned embryos, values from each group of sections from an individual embryo were averaged (2–4 sections for each sample). All statistics were performed in Graphpad Prism (version 9). Normality was assessed with a D’Agostino and Pearson omnibus test. For two groups, unpaired comparisons were made using either unpaired two-tailed *t*-tests (for normal distributions) or Mann–Whitney tests (abnormal distributions). Sample sizes, raw data, and statistical details are available in figure legends and table form in Supplementary data.

### Vesicular gamma-aminobutyric acid transporter and gephyrin puncta quantification

Puncta were quantified from three-dimensional surface projections in Imaris x64 (v9.9.1; Oxford Instruments) from confocal *z*-stacks captured with identical settings and *z*-depth (16.9 μm depth, 89 planes per transverse section). Researchers were blind to genotype throughout the surface-generating process. First, fluorescent channels underwent deconvolution. Next, surfaces were rendered beginning with a morphological split based on average puncta width of 0.4 μm, without smoothing. Next, an automatic background subtraction was applied that was toggled by the user to reflect input fluorescence. Surfaces were then generated using the maximum quantity of seed points of 0.3 μm width and then puncta were filtered at a lower area threshold of 0.16 μm^2^, based on previous literature (Fantuzzo et al., 2017). Resultant surfaces were compared with original fluorescence to ensure proper fit. Puncta outside of the spinal cord were manually removed. Area (*x* by *y* planes) measurements for vGAT and Gephyrin were recorded, as well as the center plane area of each spinal cord for normalization. Finally, the apposition of synaptic puncta in each section was calculated by filtering for vGAT-Gephyrin and Gephyrin-vGAT puncta within a proximity <250 nm (Dani et al., 2010; Shen et al., 2020), which was also normalized to the relative size of the spinal cord.

## Results

### Fmrp restricts the production of ventrolateral GABAergic interneurons

**FIGURE 1:**
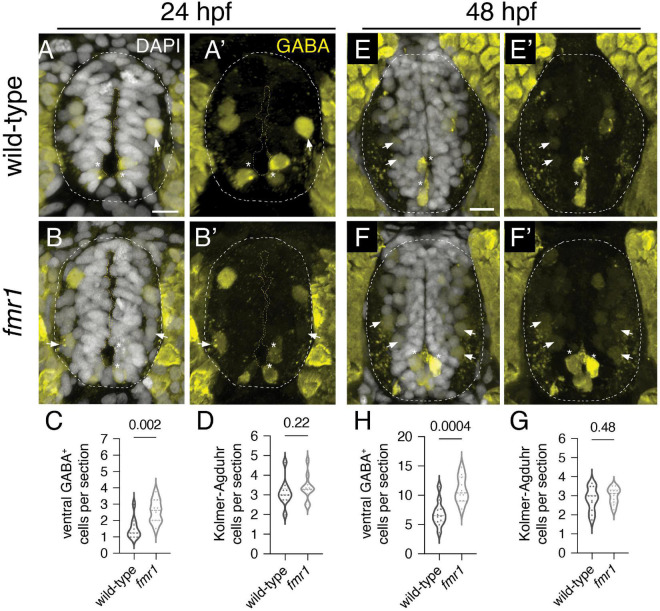
Fmrp restricts the production of GABAergic cells in the ventral spinal cord. Immunohistochemistry to detect gamma-aminobutyric acid (GABA) at 24 h post-fertilization [hpf; **(A,B)** and **(A’,B’)**] and 48 hpf **(E,F)** and **(E’,F’)** on transverse sections of trunk spinal cord reveals two populations of ventral GABA^+^ interneurons (INs): robustly GABA-expressing Kolmer–Agduhr (KA) INs (asterisks) that line the central canal (outlined in yellow dashed line) and a group of more weakly-expressing GABA^+^ INs positioned in the ventrolateral spinal cord (arrowheads). This GABA antibody produces a bright artifact outside of the spinal cord, and the cord is therefore outlined in a dashed oval in DAPI-merged images. Quantification of ventral GABA^+^ cells at 24 hpf [**(C)**; two-tailed *t*-test] and 48 hpf [**(G)**; two-tailed *t*-test], and KA neurons at 24 hpf [**(D)**; Mann–Whitney test] and 48 hpf [**(H)**; two-tailed *t*-test]. Quantification reflects the average number of cells per section, averaged by embryo. Scale bar = 10 μm. *P-values* indicated in graphs, where *p* < 0.05 is considered significant.

We previously showed that Fmrp regulates the proportional production of two essential cell types in the ventral spinal cord: oligodendrocyte lineage cells and MNs (Doll et al., 2021). Given changes in GABAergic neurotransmission in FXS (El Idrissi et al., 2005; Kratovac and Corbin, 2013), we next examined GABA INs, which provide crucial input to MNs to modulate muscle contractions and achieve coordinated locomotion (Saint-Amant and Drapeau, 2001; Higashijima et al., 2004b; Callahan et al., 2019). We used IHC on transverse trunk spinal cord sections to detect embryonic GABAergic INs, finding two main classes of cells in the ventral spinal cord: (1) brightly labeled Kolmer–Agduhr (KA) INs that innervate the central canal and are associated with chemosensory function (asterisks, Figures 1A,B,E,F; Seredick et al., 2014; Andrzejczuk et al., 2018); (2) dimly labeled GABA^+^ cells in the ventrolateral cord (arrows, Figures 1A,B,E,F). There was a 33% increase at 24 hpf and 37% increase at 48 hpf in ventrolateral GABA^+^ INs in *fmr1* embryos (Figures 1C,G) but no change in KA IN quantity at both 24 and 48 hpf (Figures 1D,H).

### Fmrp promotes expression of inhibitory synaptic proteins in embryogenesis

**FIGURE 2:**
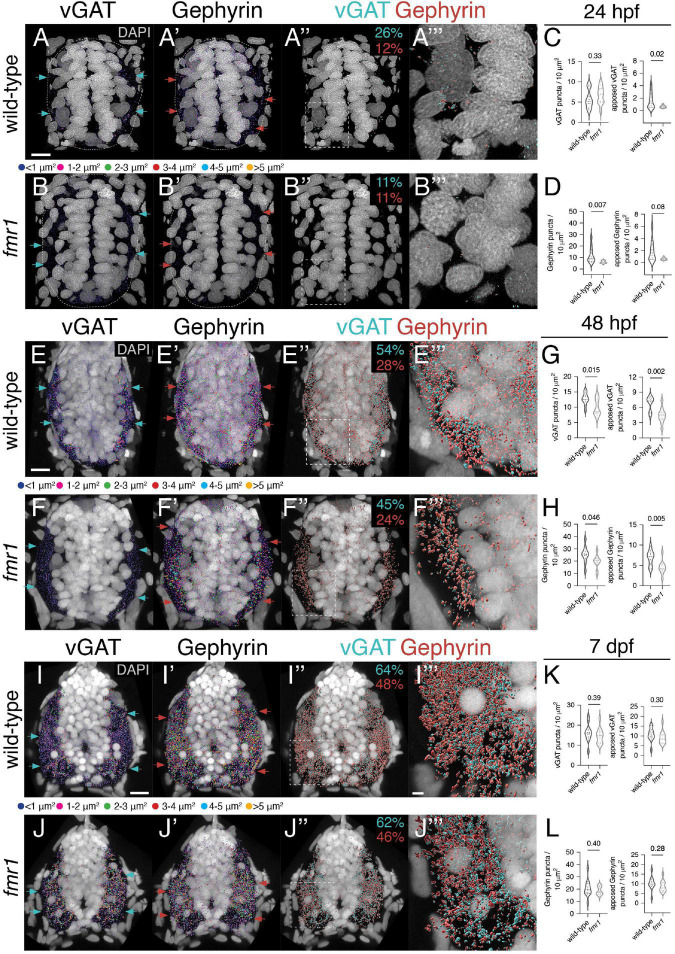
Fmrp developmentally regulates the expression of inhibitory synaptic proteins. Representative images from immunohistochemistry experiments to detect the vesicular gamma-aminobutyric acid (GABA) transporter [vesicular GABA transporter (vGAT); **(A,B,E,F,I,J)**] and the postsynaptic scaffold Gephyrin **(A’,B’,E’,F’,I’,J’)** on transverse trunk spinal cord sections of wild-type and *fmr1* mutants at 24 hpf **(A,B)**, 48 hpf **(E,F)**, and 7 dpf **(I,J)**. vGAT (cyan arrows) and Gephyrin expression (red arrows) is most concentrated in the lateral axon tracts. Apposition of vGAT and Gephyrin is shown in third panels **(A”,B”,E”,F”,I”,J”)**, which represent vGAT puncta positioned <250 nm to Gephyrin puncta, and vice versa. Quantification of vGAT **(C,G,K)** and Gephyrin puncta **(D,H,L)** normalized to spinal cord area as well as the relative amount of vGAT or Gephyrin apposed to Gephyrin or vGAT, respectively. Cyan numbers in third panels represent the percentage of vGAT puncta apposed to Gephyrin to total vGAT, while red numbers represent the percentage of Gephyrin puncta apposed to vGAT to total Gephyrin. Forth panels **(A”’,B”’,E”’,F”’,I”’,J”’)** represent a zoom of synaptic protein apposition images (indicated by dashed boxes in third panels). The puncta size color code applies to the first and second panels, not apposition data. Dashed ovals in panel **(A–B’)** denote the edges of the spinal cord at 24 hpf. Scale bars = 10 μm (2 μm for apposition zoom panels). Aside from a Mann–Whitney test to compare 24 hpf Gephyrin apposition, all other statistical comparisons from unpaired *t*-tests. *P-values* indicated in graphs, where *p* < 0.05 is considered significant. See also Supplementary Figures 1, 2.

As Fmrp also plays crucial roles in the differentiation of neurons and glia (Luo et al., 2010; Edens et al., 2019; Doll et al., 2021; Raj et al., 2021), it is possible that the excess GABAergic cells in *fmr1* mutants are not terminally differentiated and do not form synapses. To help address this possibility, we examined expression of the vesicular GABA transporter (vGAT), the presynaptic vesicular transporter for both glycine and GABA (Chaudhry et al., 1998), and the postsynaptic scaffold Gephyrin, which is crucial for the clustering and stabilization of GABAergic and glycinergic receptors (Essrich et al., 1998; Feng et al., 1998). We detected both synaptic proteins on transverse sections of embryonic (24 and 48 hpf) and larval (7 dpf) trunk spinal cord (Figure 2; Marisca et al., 2020). We next used surface modeling to quantify the density and size of vGAT^+^ and Gephyrin^+^ puncta at each developmental time point (Supplementary Figure 1), focusing on the density and apposition (pre- to post-synapse and vice versa) of synaptic proteins within the spinal cord. Fmrp did not appear to dramatically influence the relative punctal size of either protein on a global level at any time point (Supplementary Figure 2). In general, we found that the size of vGAT and Gephyrin puncta increased over development at roughly equivalent rates in wild-type and *fmr1* (Supplementary Figures 2D,E), which is indicative of synaptic maturation (Craig et al., 1996).

At 24 hpf, total vGAT density was comparable between genotypes, but significantly fewer vGAT puncta in *fmr1* were apposed to Gephyrin (Figure 2C). Gephyrin density was also reduced in *fmr1* compared to controls, including Gephyrin puncta apposed to vGAT (Figure 2D). Overall, 26% of vGAT puncta in wild-type and 11% of *fmr1* puncta were within 250 nm of a Gephyrin puncta. At the late embryonic stage (48 hpf), synaptic density was much greater in both genotypes, but *fmr1* mutants showed significant reductions in both total and apposed vGAT (Figure 2G) and Gephyrin (Figure 2H) compared to wild-type. The proportion of apposed vGAT puncta also increased dramatically in both genotypes, as 54% of wild-type and 45% of *fmr1* puncta were near Gephyrin, indicative of substantial synapse formation between 24 and 48 hpf. Finally, at larval stages we found that the density of both vGAT and Gephyrin puncta was comparable between genotypes, including the total quantity per unit area and the relative apposition of both synaptic markers (Figures 2K,L). Taken together, this data suggests that GABAergic and/or glycinergic synapse formation is hindered in *fmr1* mutants during embryonic spinal development.

### Production of glutamatergic interneurons is unchanged in *fmr1* mutants

**FIGURE 3:**
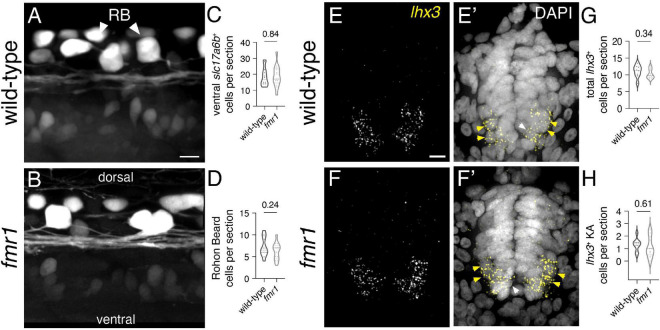
Fmrp does not regulate glutamatergic cell production. Representative lateral images of the spinal cord of live wild-type **(A)** and *fmr1* mutant **(B)** embryos expressing *slc17a6b*:LoxP-DsRed-LoxP-EGFP, a glutamatergic neuron reporter, at 24 hpf. Quantification of *slc17a6b*^+^ cells in the ventral spinal cord [**(C)**; two-tailed *t*-test], and *slc17a6b*^+^ Rohon–Beard (RB) cells in the dorsal cord [**(D)**; two-tailed *t*-test]. RBs have large cell bodies and brightly express the reporter [arrowheads, **(B)**]. Representative images of fluorescent *in situ* hybridization (FISH) experiments to detect *lhx3* expression in transverse trunk spinal cord sections of wildtype **(E)** and fmr1 **(F)** embryos at 24 hpf. Presumptive *lhx3*^+^ V2a interneurons (INs) in the lateral region are indicated with yellow arrowheads and *lhx3*^+^ KA neurons adjacent the central canal are labeled by white arrowheads **(E’,F’,E”,F”)**. Quantification of total *lhx3*^+^ cells [excluding KA; **(G)**; two-tailed *t*-test] and *lhx3*^+^ KA cells per section [**(H)**; two-tailed *t*-test]. *lhx3* quantification reflects the average number of cells per section, averaged by embryo. Scale bars = 10 μm. *P-values* indicated in graphs, where *p* < 0.05 is considered significant. See also Supplementary Figure 3.

Given the reduction in neural patterning we previously showed in *fmr1* embryos (Doll et al., 2021), it is possible that Fmrp regulates the specification of additional spinal interneurons. Glutamate is the principal excitatory neurotransmitter in the mature spinal cord (Kudo and Yamada, 1987), and glutamatergic INs are specified from several spinal progenitor domains (Higashijima et al., 2004b; Park et al., 2004). To broadly test whether Fmrp also regulates glutamatergic cell production, we used the transgenic reporter *Tg(slc17a6b:EGFP)*, as *slc17a6b* encodes the vesicular glutamate transporter (Miyasaka et al., 2009). *slc17a6b* is expressed two distinct groups of cells in the spinal cord at 24 hpf, including large clusters of cells with small somata in the ventral cord and large Rohon–Beard sensory neurons in the dorsal region (Figures 3A,B; Higashijima et al., 2004a). There was no change in the average number of EGFP^+^ ventral cells or Rohon–Beard neurons in *fmr1* mutants compared to wild-type control (Figures 3C–D), which suggests that Fmrp does not regulate the overall production of glutamatergic INs.

Embryonic spinal neuron subtypes are defined by unique genetic profiles that are initiated in specific progenitor domains (Park et al., 2004; Seredick et al., 2014; Andrzejczuk et al., 2018). We also examined a specific population of glutamatergic INs in the ventral spinal cord that are specified in the p2 domain and express the transcription factor *lhx3* (Seredick et al., 2014). V2a cells are ipsilaterally projecting INs that drive swimming behavior in larval stages (Kimura et al., 2013), and could therefore contribute to early motor behavior. We used FISH to detect *lhx3* expression in wild-type and *fmr1* mutants, finding that expression was limited to cells in the ventral half of the spinal cord, including a small population of *lhx3*^+^ cells abutting the central canal that appeared to be KA neurons (white arrowheads, Figures 3E’,F’; Supplementary Figure 3), as well as ostensible V2a INs in the ventrolateral regions of the cord (yellow arrowheads, Figures 3E,F). There was no change in the quantity of either *lhx3*^+^ subtype in *fmr1* mutants compared to wild-type (Figures 3G,H). Taken together, our results indicate no clear changes in spinal glutamatergic neuron specification in *fmr1* mutants.

### Excess ventral lateral descending interneurons are produced in the absence of Fmrp

**FIGURE 4:**
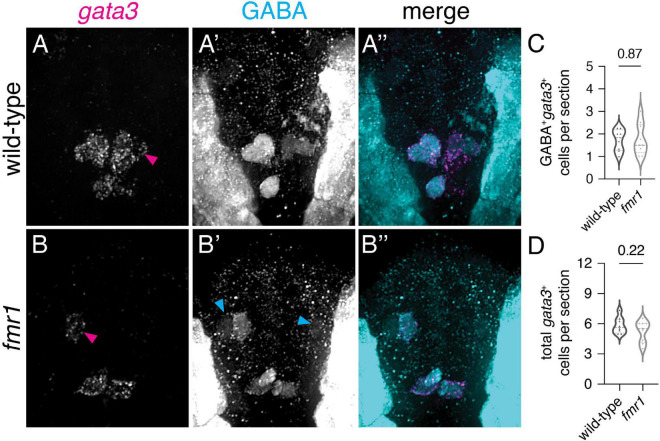
Excess GABAergic neurons in *fmr1* embryos are not specified in the p2 progenitor domain. Representative images showing fluorescent RNA *in situ* hybridization to detect *gata3* transcript in wild-type **(A)** and *fmr1* mutant embryos **(B)** alongside immunohistochemistry for gamma-aminobutyric acid (GABA) **(A’,B’)** and merged images **(A”,B”)** at 24 hpf. Quantification of GABA^+^*gata3*^+^ V2b cells [**(C)**; two-tailed *t*-test] and total *gata3*^+^ cells at 24 hpf [**(D)**; two-tailed *t*-test]. GABA^+^*gata3*^+^ V2b neurons marked with magenta arrows **(A,B)**, GABA^+^*gata3*^–^ cells indicated with cyan arrows **(B’)**. Quantification reflects the average number of cells per section, averaged by embryo. Scale bar = 10 μm. *P-values* indicated in graphs, where *p* < 0.05 is considered significant. See also Supplementary Figure 3.

We used additional fate mapping approaches to determine the origin and identity of excess GABAergic INs in *fmr1* mutants. Based on the relative position of these cells in the cord, we reasoned they could derive from either the p2 or pMN domains (Park et al., 2004; Kimura et al., 2008; Andrzejczuk et al., 2018). We first tested the p2 origin hypothesis by examining gene expression specific to GABAergic V2b INs, which express the transcription factor *gata3* (Seredick et al., 2014). We detected *gata3 via* FISH and then used IHC to label GABAergic neurons in both wild-type and *fmr1* mutants at 24 hpf, finding a few *gata3*^+^GABA^+^ cells per section that were close to the lateral edges of the cord (presumptive V2b INs, magenta arrowheads, Figures 4A,B; Supplementary Figure 3), and many *gata3*^+^GABA^+^ KA INs adjacent the central canal (Figures 4A’,B’,A”,B”). There was no difference in *gata3*^+^GABA^+^ V2b cells (Figure 4C) or total *gata3*^+^ cells (Figure 4D) in *fmr1* embryos compared to wild-type controls, though we did see excess GABA^+^*gata3*^–^ cells in *fmr1* (cyan arrows, Figure 4B’).

**FIGURE 5:**
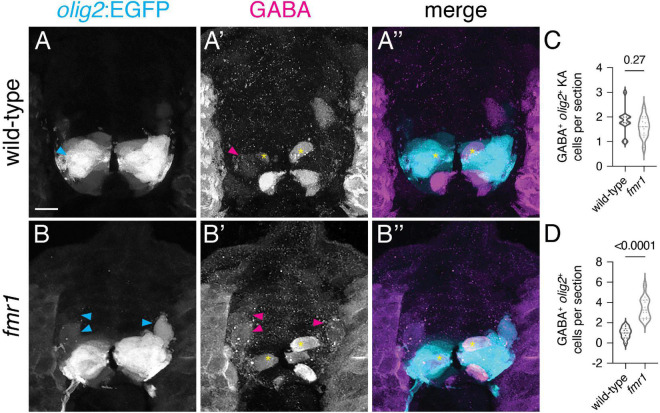
Surplus ventrolateral GABAergic neurons in *fmr1* embryos are specified in the pMN progenitor domain. At 24 h post-fertilization, there are two populations of pMN-derived GABA^+^*olig2*^+^ cells in the ventral spinal cord, as shown through immunohistochemistry for gamma-aminobutyric acid (GABA) on transverse trunk spinal cord sections of *Tg(olig2:EGFP)* embryos: dorsal KA INs [yellow asterisks, **(A’,A”,B’,B”)**] and ventral lateral descending (VeLD) INs [cyan and magenta arrowheads, **(A,A’,B,B’)**]. Quantification of KA INs [**(C)**; Mann–Whitney test] and VeLD INs [**(D)**; two-tailed *t*-test] in wild-type and *fmr1* embryos. Quantification reflects the average number of cells per section, averaged by embryo. Scale bar = 10 μm. *P-values* indicated in graphs, where *p* < 0.05 is considered significant.

As an alternative hypothesis for the origin of surplus GABA^+^ INs in *fmr1* mutants, we addressed IN specification from the pMN domain, which gives rise to a wide variety of cell types: cholinergic motor neurons, oligodendroglia, glutamatergic INs, a subpopulation of KA INs (dorsal KAs, as opposed to KAs derived from ventral p3 domain), and GABAergic VeLD INs (Park et al., 2004). We used IHC to detect GABA expression on trunk spinal cord sections of *olig2:*EGFP transgenic embryos, a pMN domain reporter (Figures 5A,B). Although there was no change in pMN-derived GABA^+^ KA cells (Figure 5C), there was a significant increase in ventrolateral *olig2*^+^GABA^+^ cells (Figure 5D). Taken together with data in Figure 1, increases shown in Figures 1C, 5D are proportional, indicating that excess GABAergic neurons in *fmr1* mutants are specified in the pMN domain.

### Fmrp regulates neurotransmitter expression in early born *mnx1*^+^ cells

**FIGURE 6:**
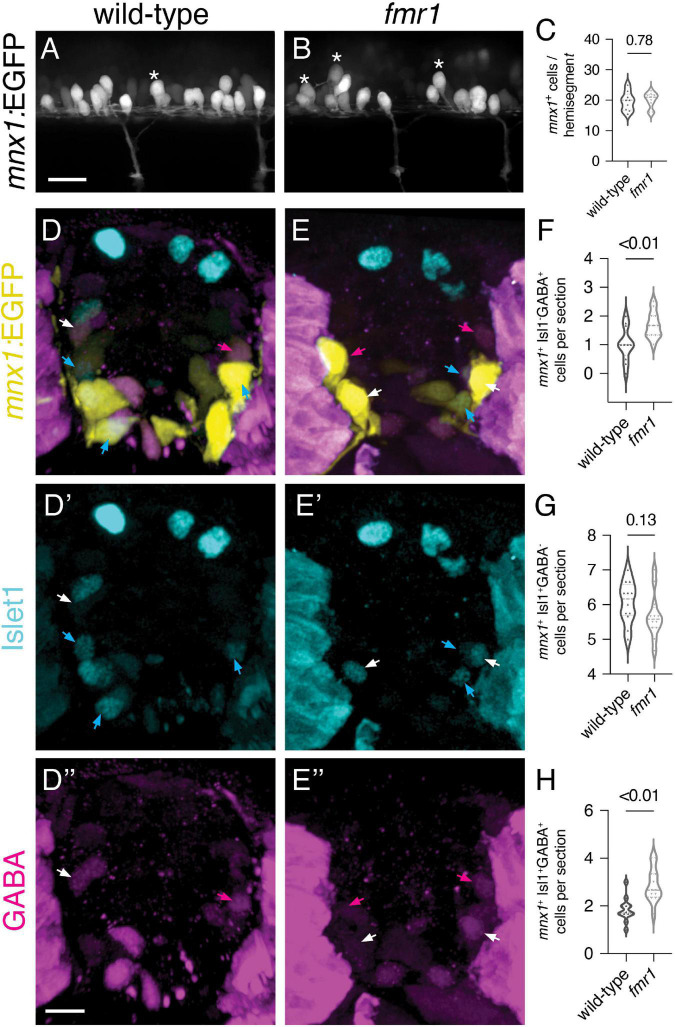
Increased early born ventral lateral descending (VeLD interneurons and GABAergic motor neurons of *fmr1* embryos **(A,B)** Live lateral images of trunk spinal cord from embryos expressing *mnx1*:EGFP, a reporter of primary motor neurons and VeLD interneurons. Asterisks indicate presumptive VeLD interneurons, with large soma situated dorsal and rostral to primary motor neurons. **(C)** Quantification of the average number of EGFP^+^ cells per hemisegment at 24 hpf. Representative images of immunohistochemistry to detect gamma-aminobutyric acid (GABA) and Islet1 on transverse trunk spinal cord sections of *mnx1:*EGFP embryos at 24 hpf. *mnx1* + cells **(D,E)** expressed a combination of the motor neuron marker Islet1 **(D’,E’)** and GABA **(D”,E”)**. Quantification of *mnx1*^+^Isl1^–^GABA^+^ presumptive VeLD interneurons [**(F)**; magenta arrows; two-tailed *t*-test], *mnx1*^+^Isl1^+^GABA^–^ motor neurons [**(G)**; cyan arrows; two-tailed *t*-test], and *mnx1*^+^Isl1^+^GABA^+^ motor neurons [**(H)**; white arrows; two-tailed *t*-test]. Quantification reflects the average number of cells per section, averaged by embryo. Scale bars = 10 μm. *P-values* indicated in graphs, where *p* < 0.05 is considered significant.

Finally, we used the *mnx1:*EGFP transgenic reporter to provide both spatial and quantitative readouts of how Fmrp regulates the development of early-born MNs and VeLD INs (Flanagan-Steet et al., 2005). At 24 hpf, *mnx1:*EGFP is expressed in clusters of primary motor neurons and VeLD interneurons that are stereotypically arranged in each hemisegment of the spinal cord (Figures 6A,B). VeLDs are positioned slightly dorsal to MNs at the rostral end of each hemisegment in the early embryonic cord of wild-type embryos, with large oval cell bodies (Figure 6A, asterisks; Seredick et al., 2012). We quantified total *mnx1*-expressing cells in live transgenic animals at 24 hpf and found no change in *fmr1* compared to wild-type controls (Figure 6C).

To further test whether Fmrp regulates differentiation of *mnx1*^+^ cell subtypes, we next detected expression of GABA and Isl1–a marker of motor neurons–on transverse trunk sections of *mnx1:*EGFP embryos at 24 hpf. We quantified cells expressing combinations of the three markers, and again found an increase in *mnx1*^+^GABA^+^Isl1^–^ presumptive VeLD INs (Figure 6F). We also saw a slight reduction in *mnx1*^+^GABA^–^Isl1^+^ MNs in *fmr1* at 24 hpf (*p* = 0.13; Figure 6G), which is in line with our previous work showing reduced Isl1^+^ MNs in *fmr1* embryos at the 20 somite stage (Doll et al., 2021). We were also surprised to find that a subset of *mnx1*^+^Isl1^+^ MNs also expressed GABA at this embryonic stage, and significantly more *mnx1*^+^Isl1^+^GABA^+^ cells were detected in *fmr1* than wild-type (Figure 6H). Taken together, this data suggests that Fmrp regulates the differentiation of early born ventral neurons.

## Discussion

The embryonic zebrafish spinal cord contains a simple, well-mapped network of spinal neurons that act in concert to facilitate the progressive development of swimming motion (Kuwada et al., 1990; Eisen, 1991; Granato et al., 1996). Motor neuron function, as the output of the locomotive circuit, is clearly crucial to the process. However, primary motor neuron subunits are produced as discrete islands in individual hemisegments (Seredick et al., 2012), which must be coordinated to produce locomotive output. Integrating interneurons provide an orchestrating influence in the generation of complete movements by linking motor neurons throughout the spinal cord (Saint-Amant and Drapeau, 2001; Warp et al., 2012). We hypothesize that surplus VeLD INs in *fmr1* embryos may drive hyperexcitable motor behavior that persists in larval and adult stages (Kim et al., 2014; Shamay-Ramot et al., 2015). This builds upon a hypothesis that VeLD interneurons synchronize motor output in the zebrafish spinal cord to drive the earliest spontaneous movements (Warp et al., 2012). In the future we plan to directly test roles for VeLDs in the initiation of embryonic motor behavior.

It remains unclear whether VeLD function is linked to the initiation of hyperexcitability in *fmr1* mutants, and if the mechanism involves GABAergic neurotransmission. The earliest spontaneous motor activity in zebrafish occurs around 22 hpf and is largely insensitive to GABAergic antagonism (though glycine appears to exert some influence; Saint-Amant and Drapeau, 2001, 2000; Downes and Granato, 2006). It appears more likely that electrical coupling through gap junctions underlies interneuron-driven entrainment of spinal neurons (Saint-Amant and Drapeau, 2001; Warp et al., 2012). In line with this, we found that expression of presynaptic vGAT and postsynaptic Gephyrin was sparse at 24 hpf as compared to later developmental time points (Figure 2). Expression of both synaptic proteins expanded dramatically in the subsequent 24 h of development, which suggests the formation of the inhibitory spinal network occurs in this critical window. It is still unknown precisely when GABA neurotransmission initiates in the cord, whether initial GABAergic signaling is depolarizing vs. hyperpolarizing and the relative weight of this neurotransmission on embryonic motor function.

The reduced expression and apposition vGAT and Gephyrin in *fmr1* mutants at the end of embryogenesis is suggestive of reduced inhibitory synapse formation (Figure 2). This stage is predicted to correspond to the developmental chloride-based polarity shift, when GABA reception becomes hyperpolarizing (Reynolds et al., 2008). At a larval stage (7 dpf), the relative density of vGAT and Gephyrin was comparable between genotypes, which may indicate recovery of inhibitory synapse formation. However, this GABA polarity transition is delayed in FXS model mice (He et al., 2014), and *fmr1* zebrafish remain hyperexcitable at larval and adult stages (Kim et al., 2014; Shamay-Ramot et al., 2015). Our immunohistochemistry data is only a proxy of synapse formation and the involvement and intersection of Fmrp and GABA in motor excitability will require a careful cell type-specific dissection of neuronal physiology. We also plan to use transgenic reporters to examine the subcellular distribution of synaptic protein expression in specific classes of neuronal subtypes.

Ultimately, FXS pathology is rooted in molecular mechanisms due to the loss of the RNA binding protein FMRP. FMRP is highly expressed in neurons during early development and declines with age (Bonaccorso et al., 2015). In addition, it is increasingly clear that FMRP function in stem/progenitor cells provides crucial regulation of the specification and differentiation of unique cell types (Tervonen et al., 2009; Edens et al., 2019; Doll et al., 2021; Raj et al., 2021; Zhang et al., 2022). Our work shows that Fmrp regulates the differentiation and neurotransmission profiles of ventral spinal neurons (Figure 5), with increased production of VeLD INs and GABAergic primary motor neurons (Figure 6). In adult zebrafish, a minority of spinal cholinergic motor neurons co-express GABA (∼7%; Pedroni and Ampatzis, 2019), which are positioned in comparable regions of the cord, though it remains unclear what roles GABA is playing in these cells. As the total density of *mnx1*-expressing cells was unchanged in *fmr1* embryos, Fmrp appears to also regulate the proportionate production of neuronal subtypes. In related studies, disruption of Isl2 function or loss of *islet1* also lead to changes in pMN cell fate, such that VeLD-like cells are overproduced at the expense of primary motor neurons (Segawa et al., 2001; Moreno and Ribera, 2014). We also plan to characterize the genetic signatures of pMN progenitors and identify Fmrp target mRNAs in pMN-derived cells to help reveal the mechanisms that underlie proportionate cell production and maturation during motor circuit development.

## Data availability statement

The original contributions presented in this study are included in the article/Supplementary material, further inquiries can be directed to the corresponding author.

## Ethics statement

The animal study was reviewed and approved by the Institutional Animal Care and Use Committee at the University of Colorado School of Medicine, this study follows the US National Research Council’s Guide for the Care and Use of Laboratory Animals, the US Public Health Service’s Policy on Humane Care and Use of Laboratory Animals, and Guide for the Care and Use of Laboratory Animals.

## Author contributions

CB: formal analysis and investigation. KM: investigation. CD: conceptualization, formal analysis, investigation, writing, and visualization. All authors contributed to the article and approved the submitted version.
