# Supplementary material for: Fmrp regulates neuronal balance in embryonic motor circuit formation
Source: Front Neurosci. 2022 Nov 3;16:962901. doi: 10.3389/fnins.2022.962901 (PMC9669763; doi:10.3389/fnins.2022.962901)
Supplement: Supplementary file 7 [file Table_1.DOCX]

Statistics

| **Figure 1** |  | **wild-type** | ***fmr1*** | test |
| --- | --- | --- | --- | --- |
| GABAergic cell quantification | 24 hpf ventral GABA^+^ cells (KA excluded) | n=10 embryos (39 sections); mean=1.50±0.20 cells | n=10 embryos (38 sections); mean=2.25±0.22 cells | unpaired two-tailed t-test |
|  | 24 hpf GABA^+^ KA cells | mean=3.12±0.21 cells | mean=3.37±0.21 cells | Mann-Whitney test |
|  | 48 hpf ventral GABA^+^ cells (KA excluded) | n=11 embryos (41 sections); mean=6.86±0.64 cells | n=10 embryos (41 sections); mean=10.94±0.72 cells | unpaired two-tailed t-test |
|  | 48 hpf GABA^+^ KA cells | mean=2.83±0.19 cells | mean=3.0±0.14 cells | unpaired two-tailed t-test |
| **Figure 2** |  | **wild-type** | ***fmr1*** | test |
| Synaptic puncta  quantification | 24 hpf puncta/area quantification; Gephyrin | n=12 embryos (28  sections); mean=12.0±1.95 puncta/10 µm^2^ | n=12 embryos (31 sections); mean=6.07±0.32 puncta/10 µm^2^ | unpaired two-tailed test |
|  | 24 hpf apposed Gephyrin puncta/area | mean=1.84±0.49 puncta/10 µm^2^ | mean=0.63±0.05 puncta/10 µm^2^ | Mann-Whitney test |
|  | 24 hpf puncta/area; vGAT | mean=5.76±0.65 puncta/10 µm^2^ | mean=6.64±0.60 puncta/10 µm^2^ | unpaired two-tailed t-test |
|  | 24 hpf apposed vGAT puncta/area | mean=1.6±0.37 puncta/10 µm^2^ | mean=0.64±0.05 puncta/10 µm^2^ | unpaired two-tailed t-test |
|  | 24 hpf spinal cord area | mean=5817±142.7 µm^2^ | mean=6159±182.0 µm^2^ |  |
|  | 48 hpf puncta/area quantification; Gephyrin | n=10 embryos (27 sections); mean=25.2±2.17 puncta/10 µm^2^ | n=11 embryos (26 sections) ; mean=19.49±1.62 puncta/10 µm^2^ | unpaired two-tailed t-test |
|  | 48 hpf apposed Gephyrin puncta/area | mean=6.89±0.5 puncta/10 µm^2^ | mean=4.46±0.5 puncta/10 µm^2^ | unpaired two-tailed t-test |
|  | 48 hpf puncta/area; vGAT | mean=12.69±0.61 puncta/10 µm^2^ | mean=9.95±0.81 puncta/10 µm^2^ | unpaired two-tailed t-test |
|  | 48 hpf apposed vGAT puncta/area | mean=6.75±0.45 puncta/10 µm^2^ | mean=4.4±0.45 puncta/10 µm^2^ | unpaired two-tailed t-test |
|  | 48 hpf spinal cord area | mean=6111±219.6 µm^2^ | mean=6485±234.2 µm^2^ |  |
|  | 7d puncta/area quantification; Gephyrin | n=12 embryos (32 sections); mean=20.59±2.0 puncta/10 µm^2^ | n=12 embryos (31 sections) ; mean=18.69±0.98 puncta/10 µm^2^ | unpaired two-tailed t-test |
|  | 7d hpf apposed Gephyrin puncta/area | mean=10.19±1.0 puncta/10 µm^2^ | mean=8.76±0.83 puncta/10 µm^2^ | unpaired two-tailed t-test |
|  | 7d puncta/area quantification; vGAT | mean=16.13±1.3 puncta/10 µm^2^ | mean=14.63±1.1 puncta/10 µm^2^ | unpaired two-tailed t-test |
|  | 7d apposed puncta/area; vGAT | mean=10.72±0.99 puncta/10 µm^2^ | mean=9.27±0.95 puncta/10 µm^2^ | unpaired two-tailed t-test |
|  | 7 dpf spinal cord area | mean=6213±190.4 µm^2^ | mean=5291±129.0 µm^2^ |  |
| **Figure 3** |  | **wild-type** | ***fmr1*** | test |
| Glutamatergic interneuron quantification | 24 hpf total cells | n=12 embryos; mean=32.5±2.5 cells | n=13 embryos; total mean=31.23±2.9 cells | unpaired two-tailed t-test |
|  | ventral cells | mean=18.33±1.7 cells | mean=18.85±1.9 cells | unpaired two-tailed t-test |
|  | Rohan-Beard cells | mean=7.25±0.64 cells | mean=6.23±0.54 cells | unpaired two-tailed t-test |
| *lhx3^+^* cell  quantification | V2a cell counts | n=8 embryos (29 sections); mean=11.06±0.66 cells | n=9 embryos(26 sections); total mean=10.28±0.47 cells | unpaired two-tailed t-test |
|  | KA cell counts | mean=1.31±0.19 cells | mean=1.13±0.28 cells | unpaired two-tailed t-test |
| **Figure 4** |  | **wild-type** | ***fmr1*** | test |
| p2 domain fate mapping | 24 hpf  total *gata3*^+^ cells | 8 embryos (29 sections); mean=5.83±0.29 cells | 9 embryos (26 sections); mean=5.24±0.35 cells | unpaired two-tailed t-test |
|  | 24 hpf  *gata3*^+^GABA^+^ cells | 8 embryos (29 sections); mean=1.72±0.17 cells | 9 embryos (26 sections); mean=1.77±0.23 cells | unpaired two-tailed t-test |
| **Figure 5** |  | **wild-type** | ***fmr1*** | test |
| pMN GABA cells | *olig2*^+^GABA^+^ KA’ cells | n=11 embryos (51 sections); mean=1.82±0.17 cells | n=12 embryos (64 sections); mean=1.59±0.12 cells | Mann-Whitney test |
|  | *olig2*^+^GABA^+^ non-KA cells | mean=1.02±0.16 cells | mean=3.47±0.31 cells | unpaired two-tailed t-test |
|  | total GABA^+^ cells (KA excluded; not shown) | mean=3.79±0.22 cells | mean=5.86±0.31 cells | Mann-Whitney test; p<0.0001 |
|  | ventral GABA^+^ cells (KA excluded; not shown) | mean=1.14±0.1 cells | mean=2.23±0.2 cells | unpaired two-tailed t-test; p<0.01 |
| **Figure 6** |  | **wild-type** | ***fmr1*** | test |
| *mnx1:*EGFP^+^ cell quantification  (VeLD) | 24 hpf,  *mnx1*^+^Islet^-^GABA^+^  cells | n=12 embryos; mean=1.02±0.17 cells | n=12 embryos; mean=1.68±0.13 cells | unpaired two-tailed t-test; p<0.01 |
| Isl1^+^ motor neurons | *mnx1*^+^Isl1^+^GABA^-^  cells (non-KA) | mean=6.06±0.17 cells | mean=5.67±0.19 cells | unpaired two-tailed t-test; p=0.13 |
| Isl1^+^ motor neurons (GABA^+^) | *mnx1*^+^Isl1^+^GABA^+^  cells (non-KA) | mean=1.84±0.14 cells | mean=2.86±0.21 cells | unpaired two-tailed t-test; p<0.01 |
