# Supplementary figures and images for: Fmrp regulates neuronal balance in embryonic motor circuit formation
Source: Front Neurosci. 2022 Nov 3;16:962901. doi: 10.3389/fnins.2022.962901 (PMC9669763; doi:10.3389/fnins.2022.962901)

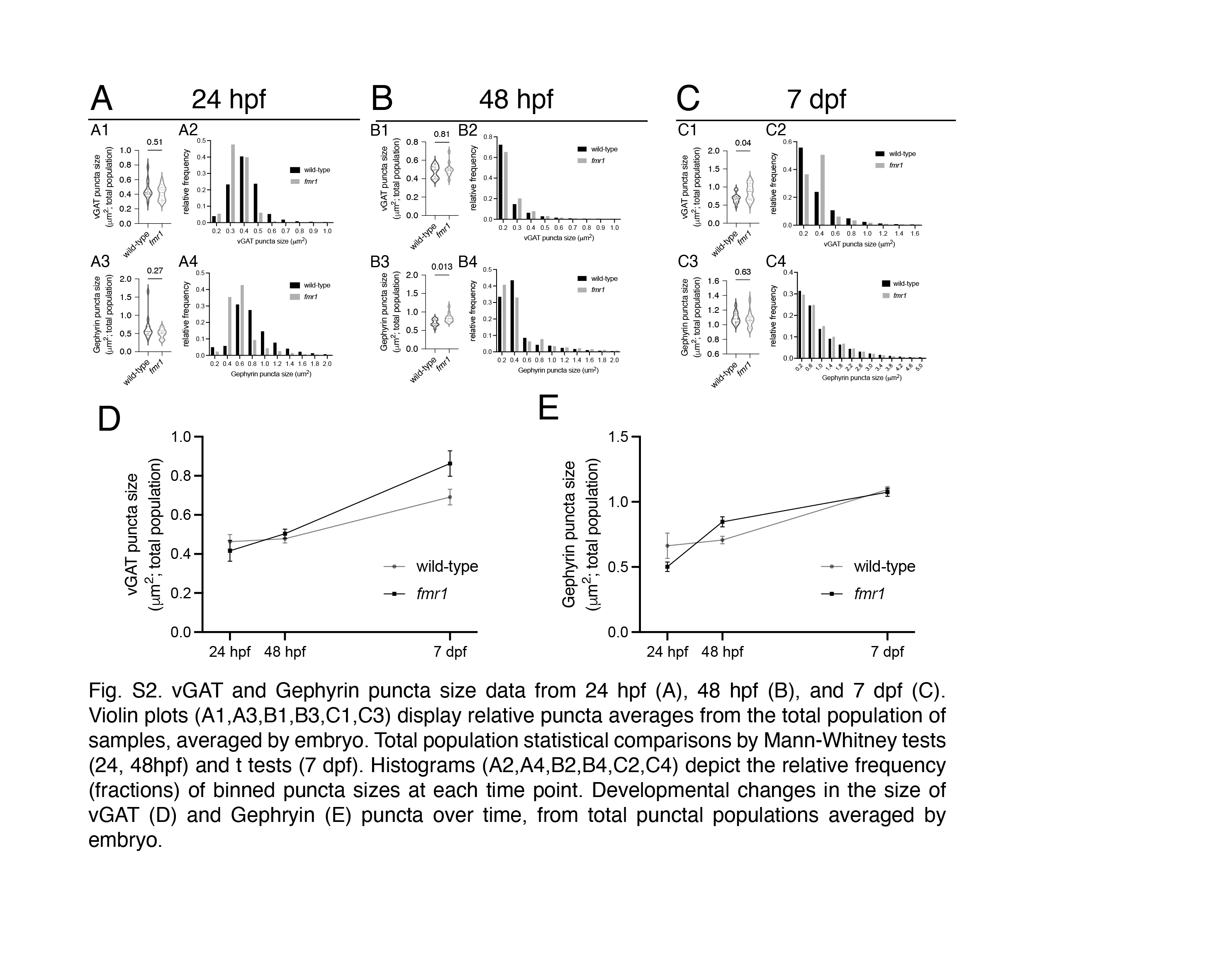

Supplement: Supplementary file 8 [file Image_1.JPEG]

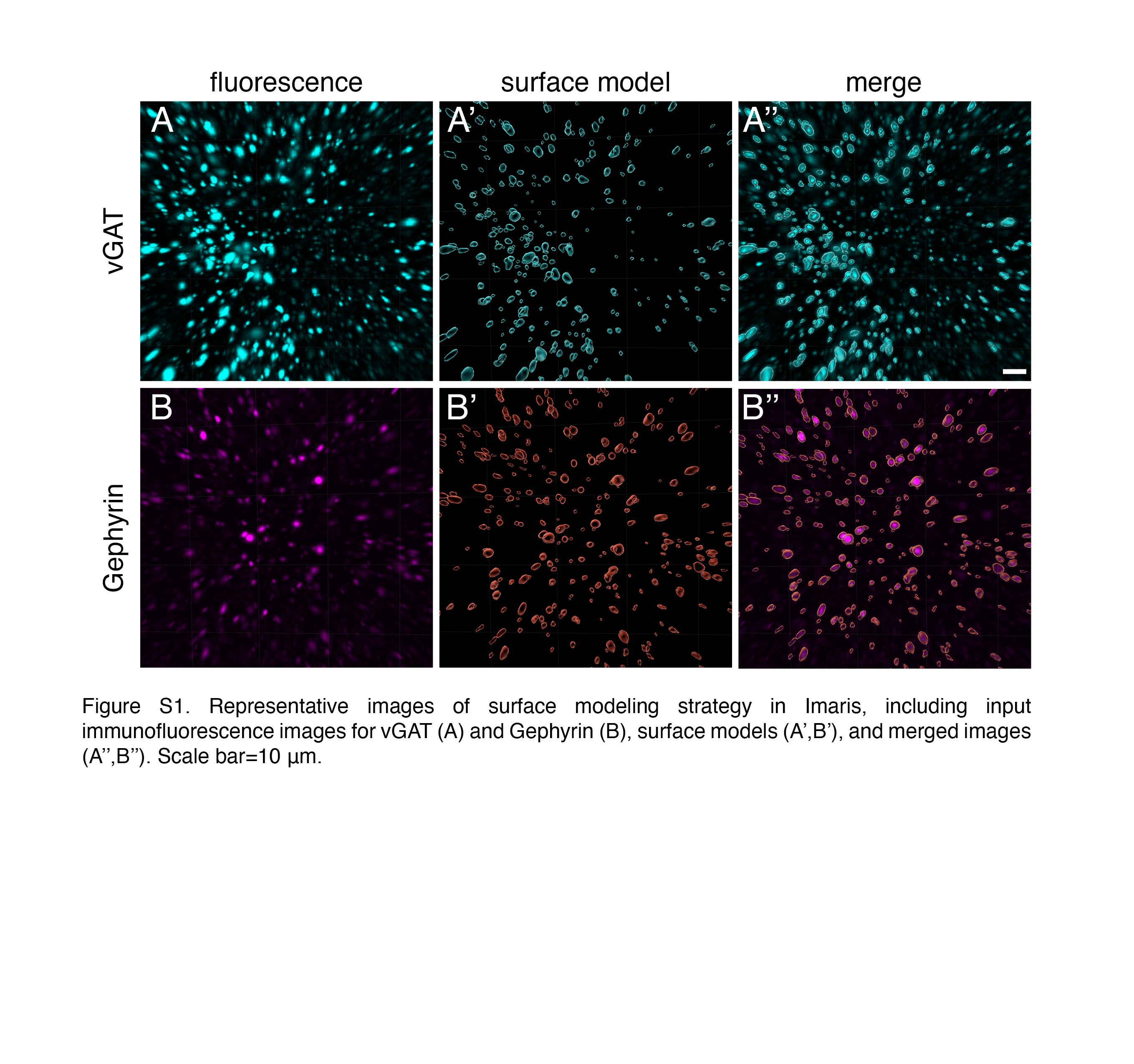

Supplement: Supplementary file 9 [file Image_2.JPEG]

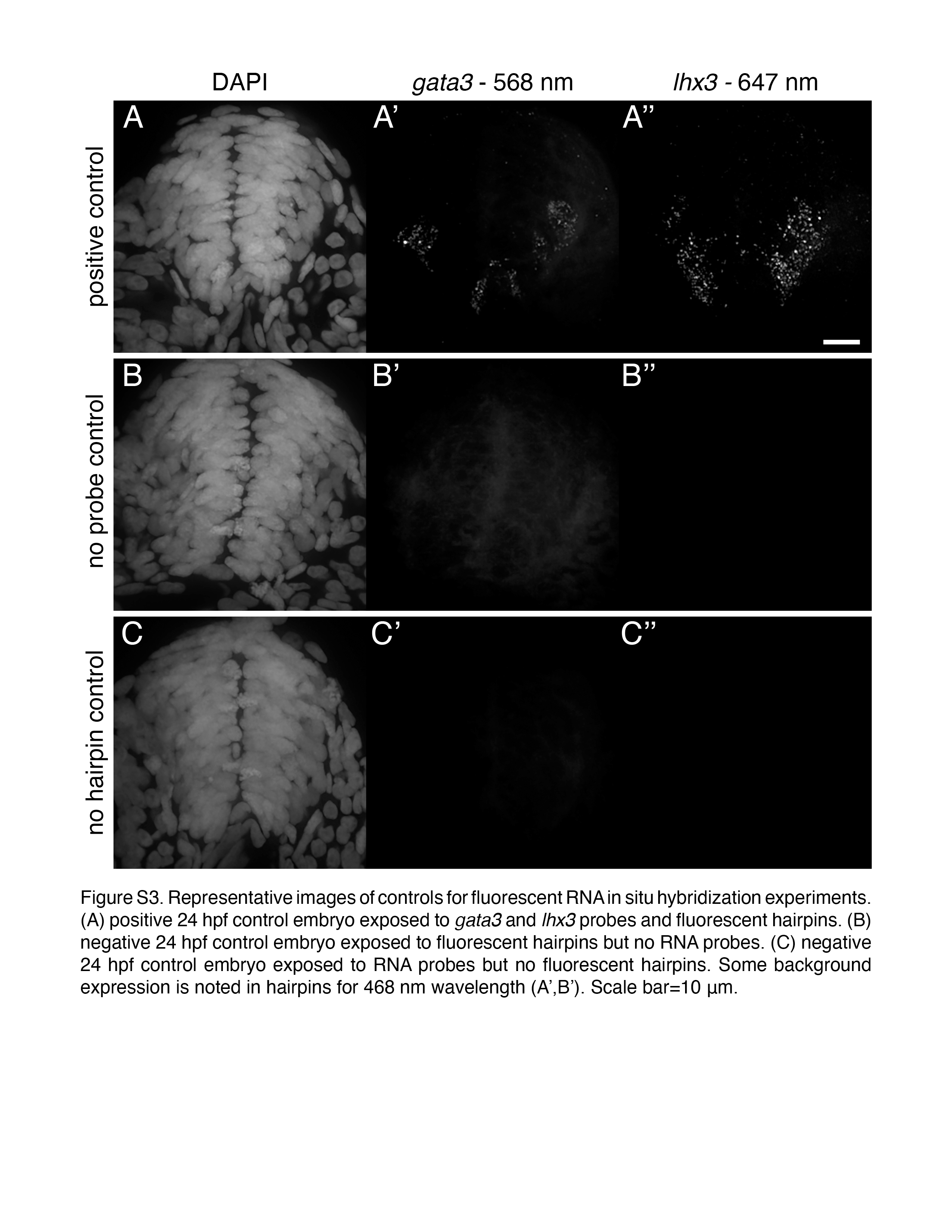

Supplement: Supplementary file 10 [file Image_3.JPEG]
